# Prostate cancer treatment costs increase more rapidly than for any other cancer—how to reverse the trend?

**DOI:** 10.1007/s13167-022-00276-3

**Published:** 2022-03-01

**Authors:** J. Ellinger, A. Alajati, P. Kubatka, F. A. Giordano, M. Ritter, V. Costigliola, O. Golubnitschaja

**Affiliations:** 1 Department of Urology, University Hospital Bonn, Rheinische Friedrich-Wilhelms-Universität Bonn, 53127 Bonn, Germany; 2 Department of Medical Biology, Jessenius Faculty of Medicine, Comenius University in Bratislava, 03601 Martin, Slovakia; 3 Department of Radiation Oncology, University Hospital Bonn, Rheinische Friedrich-Wilhelms-Universität Bonn, 53127 Bonn, Germany; 4 European Medical Association, EMA, 1160 Brussels, Belgium; 5 Predictive, Preventive and Personalised (3P) Medicine, Department of Radiation Oncology, University Hospital Bonn, Rheinische Friedrich-Wilhelms-Universität Bonn, 53127 Bonn, Germany

## Abstract

According to GLOBOCAN, about 1.41 million new prostate cancer (PCa) cases were registered in the year 2020 globally. The corresponding socio-economic burden is enormous. Anti-cancer mRNA-based therapy is a promising approach, the principle of which is currently applied for anti-COVID-19 vaccination, undergoing a detailed investigation in populations considering its short- and long-term effectiveness and potential side effects. Pragmatically considered, it will take years or even decades to make mRNA therapy working for any type of cancers, and if possible, for individual malignancy sub-types which are many specifically for the PCa. Actually, the costs of treating PCa are increasing more rapidly than those of any other cancer. The trend has to be reversed now, not in a couple of years. In general, two main components are making currently applied reactive (management of clinically manifested disease) PCa treatment particularly expensive. On one hand, it is rapidly increasing incidence of the disease and metastatic PCa as its subtype. To this end, rapidly increasing PCa incidence rates in young and middle-aged male sub-populations should be taken into account as a long-term contributor to the metastatic disease potentially developed later on in life. On the other hand, patient stratification to differentiate between non-metastatic PCa (no need for an extensive and costly treatment) and particularly aggressive cancer subtypes requiring personalised treatment algorithms is challenging. Considering current statistics, it becomes obvious that reactive medicine got at its limit in PCa management. Multi-professional expertise is unavoidable to create and implement anti-PCa programmes in the population. In our strategic paper, we exemplify challenging PCa management by providing detailed expert recommendations for primary (health risk assessment), secondary (prediction and prevention of metastatic disease in PCa) and tertiary (making palliative care to the management of chronic disease) care in the framework of predictive, preventive and personalised medicine.

## Reactive medicine is at its limit in PCa management: who is affected?

### Prostate cancer (PCa) as a socio-economic burden to the society around the globe

According to GLOBOCAN 2020, about 1.41 million new PCa cases were registered worldwide [1]. As estimated for 2021, 608,570 US Americans died from cancer corresponding to more than 1600 deaths per day; 248,530 PCa new cases (26% of all male cancers) and 34,130 PCa-related deaths were registered [2]. Similarly for Europe, PCa accounts for almost 21.8% of all newly diagnosed cancer patients and about 10% of cancer-related deaths. Keeping these statistics in mind, PCa is the leading type of tumours in 28 European countries [3].

The portion of metastatic PCa is increasing [4]. To this end, rapidly increasing PCa incidence rates in young sub-populations should be taken into account as a long-term contributor to the metastatic disease potentially developed later on in life [4]. For example, clear trends have been recorded in the UK for PCa as becoming younger over the last decades [5].

There is an evident acceleration of the metastatic cancer subtypes as demonstrated for PCa cases in the USA: compared to an annual increase of 0.58% in 2008, a strongly accelerated annual increase of 2.74% was recorded in 2012 specifically magnified for men aged below 69 years [6].

Considering the overall economic burden to the society, in 2006, 106.7–179.0 million euros (€) were dedicated to PCa management in the European countries exemplified by the UK, Germany, France, Italy, Spain and the Netherlands. In the same year, for the USA, the total estimated expenditure on prostate cancer was 9.862 billion US dollars considering $10,612 versus $33,691—the mean annual costs per patient in the initial phase after diagnosis versus corresponding costs in the last year of life, respectively. That time experts concluded that ‘Unless new strategies are devised to increase the efficiency of healthcare provision, the economic burden of prostate cancer will continue to rise’ [7]. Indeed, in the 3rd decade of the twenty-first century, the costs of treating PCa are increasing more rapidly than those of any other cancer.

### PCa management challenges: expert concerns and recommendations

#### Case 1

A 49-year-old patient presented with an increased PSA (8.9 ng/mL) in our outpatient office; he did not complain of urological problems. The digital-rectal examination and transrectal ultrasound were without suspicious findings; the prostate volume was measured at 44 mL. Multi-parametric magnetic resonance imaging (mpMRI) was planned, which showed a PI-RADS-3 lesion in the anterior transitional zone of the right mid prostate gland. The patient was therefore scheduled for an MRI/TRUS fusion biopsy of the prostate. Histologically, prostate cancer was excluded, and the patient will follow regular precautionary examinations by his general practitioner.

A major problem of PSA testing is the limited specificity. Within a prospective multicenter cohort of 6630 men, the number of PSA levels above a threshold of 4 ng/mL was 12%, and the positive predictive value of PSA for the detection of PCa in a subsequent biopsy was 31% [8]. To reduce the number of unnecessary biopsies and to improve the diagnostic specificity, improved imaging using mpMRI is recommended. A targeted biopsy allows the detection of a high number of significant cancers and the reduction of low-risk cancers [9, 10]. However, a negative MRI does not exclude PCa: in 7% of patients with a negative MRI, PCa was overlooked by MRI in the PROMIS trial, although these were usually low-grade and small-sized [11]. Thus, regular precautionary examinations are still warranted in the future. The identification of improved biomarkers is warranted to achieve a more precise follow-up and probably a reduced number of follow-up examinations.

#### Case 2

A 45-year-old patient presented with flank pain in the outpatient office. The examination was without the symptom correlation; however, digital-rectal examination showed a suspicious induration of the left prostatic lobe. Serum PSA was 0.69 ng/mL with a free PSA ratio of 33.3%. Transrectal ultrasound was unsuspicious and prostate volume was measured with 20 mL. His father died from prostate cancer. The patient refused prostate biopsy at this time. Two years later, the patient returned to our outpatient office for prostate biopsy; PSA was 0.68 ng/mL (free PSA 30.1%). The transrectal biopsy detected a Gleason score 3 + 3 = 6 prostate cancer (tumour diameter 1 mm in one biopsy core). The patient decided to go on active surveillance. The PSA increased slowly during the following 6 years up to 1.03 ng/mL. During follow-up, repeated prostate biopsies were scheduled after 12 months (without tumour), 24 months (2 mm prostate cancer area in one biopsy core, Gleason score 3 + 3 = 6), 36 months (2 mm prostate cancer area in one biopsy core, Gleason score 3 + 3 = 6), 50 months (1 mm prostate cancer area in one biopsy core, Gleason score 3 + 3 = 6) and 62 months (2 mm prostate cancer area in two biopsy cores, Gleason score 3 + 3 = 6), and demonstrated stable disease. The biopsy at 75 months demonstrated upgrading (1 biopsy: 2 mm, Gleason score 3 + 4 = 7a; 25% Gleason 4 pattern; 2 biopsies: 1 mm and 2 mm, Gleason score 3 + 3 = 6) with 3 positive biopsies in the biopsy. The mpMRI during active surveillance (performed on 50, 61 and 74 months after initial diagnosis) was always without suspicious findings. The patient thus decided to have a robot-assisted laparoscopic radical prostatectomy. The histological finding was pT2c, Gleason score 3 + 4 = 7a, R0, L0 and V0.

Active surveillance is a recommended treatment for patients with low-risk PCa. Major concerns regarding active surveillance are related to the optimal time point to start a curative to avoid locally advanced PCa or metastasis at the time of prostatectomy or radiation. Follow-up investigations are based on regular PSA measurements, digital-rectal examination, mpMRI and re-biopsies [12]. However, these modalities are imperfect, and better biomarkers are warranted. For example, the Oncotype DX Genomic Prostate Score, a 17-gene expression assay, may be helpful for decision-making when planning active surveillance. The Oncotype DX Genomic Prostate Score allowed cost-effective treatment guiding [13], but recently, a model containing the Oncotype DX Genomic Prostate Score did not improve stratification of risk for adverse pathology in addition to clinical variables [14].

#### Case 3

A 39-year-old patient was referred to our hospital with perineal pain and suspicion of prostate cancer. The PSA was 20.9 ng/ml, digital-rectal examination showed a cT4 prostate tumour. The mpMRI showed a 60 × 53 × 62 mm partially necrotic tumour with seminal vesicle infiltration, metastatic pelvic lymph nodes, and multiple osseous pelvic metastases. Prostate biopsy confirmed acinar prostate cancer (Gleason Score 4 + 5 = 9). PSMA-PET/CT diagnosed disseminated osseous metastasis. Thus, therapy with docetaxel and leuproreline acetate was initiated. After three cycles of docetaxel, clinical and radiological progression was observed. Percutaneous radiotherapy of the pelvis (45 Gy) was performed for gross hematuria. The patient was re-biopsied; histological examination showed now a squamous differentiation without the presence of residual adenocarcinoma. The molecular analysis excluded microsatellite instability, PD-L1 expression and mutations in BRCA1/2 genes; however, a pathogen mutation in the beta-catenin gene (c. 101G > T; gain-of-function mutation) was observed, as well as functionally uncharacterized mutations in the EGFR (c. 1498 + 2dup) and TP53 (c. 376-2del) mutations. Therefore, the patient underwent chemotherapy with cisplatin, 5-fluorouracil and cetuximab; however, the tumour progressed after 4 cycles and the patient died finally 8 months after the initial diagnosis.

Patients with newly diagnosed metastatic PCa are scheduled for androgen deprivation therapy in combination with docetaxel chemotherapy, abiraterone acetate, apalutamide or enzalutamide. Although there are different therapy modalities with similar outcomes, it seems reasonable that—depending on the molecular signature of the tumour—the treatment approaches may differ in their probability to achieve response. So far, there is lack of a biomarker predicting the response to medical therapy in patients with primary metastatic PCa. Recent studies in patients with castration-resistant prostate cancer (CRPC) highlighted the importance of molecular stratification in the planning of personalised therapy. The PROfound [15] and TRITON3 [16] trials demonstrated that patients with a CRPC and a mutation in BRCA1, BRCA2 or ATM benefited from olaparib or rucaparib therapy in a 2nd-line therapy setting. Furthermore, pembrolizumab was efficient in patients with CRPC with proven microsatellite instability (KEYNOTE-158 study) [17]. Thus, molecular diagnostics will gain considerable importance in the planning of medical treatment of PCa patients in the future. At this stage, however, it must be noted that the frequency of mutations in the BRCA1, BRCA2 or ATM genes is infrequent (approximately 10% of CRPC patients harbour a qualifying DNA mutation) [18]. Detection of microsatellite instability is even rarer, at approximately 3% in prostate cancer [19]. Thus, further research for improved molecular stratification is necessary to achieve personalised treatment in CRPC patients.

## Primary care is at the forefront of paradigm change from reactive to the cost-effective predictive approach

The majority of PCa cases are preventable that creates a robust platform for the paradigm change from reactive to predictive, preventive and personalised medicine [4]. PCa is a systemic multi-factorial disease resulting from an imbalanced interplay between risks and protective factors [20]. The disease is developing over years or even decades of life from a suboptimal health status characterised by a reversible damage to health, to the clinical manifestation of the disease (irreversible organ damage). A suboptimal health condition is the operating timeframe for the primary care to reverse health damage by applying health risk assessment and damage-mitigating, protective and preventive measures tailored to the individual [21]. Herewith, we exemplify tools instrumental for the primary care in overall PCa management.

### The roadmap for health risk assessment

Health risk assessment is based on individualised patient profiling. Considering its optimal cost-efficacy, primary health caregivers are recommended to apply first *specialised surveys* (see below) to consider potential health risks linked to the disease-relevant phenotypes followed by more specific/targeted *molecular characterisation of the disease predisposition* (see below) [22].

#### Specialised survey (phenotyping)

Several risk factors which per evidence are considered strong contributors to PCa initiation, development and progression can be identified by the specialised survey used, therefore for a disease-relevant phenotyping. The survey comprises information about internal and external risk factors such as the following ones:

- Cancer predisposition in the family
- Toxic environment/stress overload
- Disturbed microcirculation
- Compromised immune system
- Chronic inflammation
- Hormonal dysregulation
- Adequate physical and sexual activity
- Dietary habits/body mass index (BMI)

amongst others.

Collected information gives the clue for the follow-up molecular characterisation of individual disease predisposition, with other words, whether specific tests are essential and if yes, which ones.

#### Molecular characterisation of disease predisposition

Depending on the identified phenotype (see above “Specialised survey (phenotyping)”), the risk factor–related molecular patterns should be analysed that together represents advanced predictive diagnostics. Determination of specific biomarker patterns in body fluids, such as blood plasma/serum and tear fluid, is the most promising non-invasive approach to identify persons at high risk [4, 20]. To this end, a recently published review article demonstrated mass spectrometric analysis of tear fluid as a powerful tool to predict PCa and to discriminate between benignancy and malignancy of prostate [23]. For example, compared to many other cancers, specifically lacryglobin in tears was demonstrated as being present to 100% of patients with PCa. In the control group, several individuals stratified as non-diseased using standard diagnostic methods, demonstrated both—lacryglobin in tears and PCa history in the family; this information is important to targetly protect affected individuals against the disease development [24].

### Mitigating measures

By applying mitigating measures, one should differentiate between a generalised anti-cancer protection, which follows general principles based on population statistics available on one hand, and on the other hand, personalised prevention tailored to the individual. Corresponding dis/advantages are presented below.

#### Generalised anti-cancer protection

Generalised anti-cancer protection is a low-cost approach (that is an evident advantage) utilising general principles, such as physical activity is healthier than inactivity, high BMI is a risk factor, and sleep quality is an important protection against cancer development. However, a big disadvantage of the approach is the principle of its non-personalised character ‘one size fits all’. Indeed, many scientific projects have demonstrated that ‘healthy’ physical activity is highly individual, both high and low BMI might be an individual risk factor and individual sleep duration might be disease-relevant [25]. Therefore, personalised or targeted prevention is considered significantly more effective for individuals in suboptimal health potentially predisposed to PCa initiation and development [4, 20].

#### Personalised prevention

Personalised prevention is based on an individual profile including both phenotyping and molecular characterisation of the disease predisposition. Costs of corresponding diagnostic tools should be kept in mind as a disadvantage. Advantage is the evidence-based approach tailored to the person under treatment but not an ‘averaged individual’ considered. For the optimal cost-effective approach, an application of multi-parametric analysis is crucial to prevent PCa as a multi-factorial disease [4]. Corresponding approach can be exemplified by stress overload to mitochondria (e.g. estimated by so-called “mitochondrial health index”) and consequently excessive release of reactive oxygen species (ROS), resulting in oxidative cellular stress which prostate tissue is highly sensitive to. Associated metabolic alterations cause an imbalanced upregulation of the androgen receptor signalling which further promotes ROS in excess on one hand and on the other hand dysregulates the nuclear factor erythroid 2-related factor 2 (Nrf2) with concomitantly impaired antioxidant protection enzymatic chain (catalase, superoxide dismutase and glutathione peroxidise). Inactivation of the antioxidant gene cascade is implicated in the prostate cancer initiation but can be restored by natural antioxidant defence supplements (phytochemicals) with a potential to reverse carcinogenesis in the prostate tissue [20].

## Risk assessment, patient stratification and targeted prevention of metastatic disease are crucial for secondary PCa care

As demonstrated above, two major issues contribute to the enormous economic burden in overall PCa management, namely rapidly increasing incidence on one hand, and on the other hand, challenging patient stratification to differentiate between non-metastatic PCa (no need for an extensive treatment) and particularly aggressive cancer subtypes requiring personalised treatment algorithms. The latter is exactly the task for an advanced secondary care. At this level, implementing PPPM strategies (risk assessment, patient stratification, targeted prevention of metastatic disease and treatment algorithms tailored to the person) is crucial to save lives and to reverse currently observed economic trends.

CTCs (circulating tumour cells) tests including enumeration and molecular characterisation are instrumental for patients with clinically manifested PCa. The prognostic value of CTCs is higher compared with routinely used biomarkers (prostate-specific antigen, lactate dehydrogenase and bone-specific alkaline phosphatase); the highest C-index was achieved in the multi-parametric analysis combining conventional markers with CTCs enumeration [26]. This study demonstrated CTCs density in blood as a robust indicator at any step of the disease development and progression being a more powerful predictor compared to any other commonly used biomarkers. Finally, multiomic targets including disease- and stage-specific cell-free nucleic acid patterns are highly recommended for secondary PCa care [27]. To this end, PCa secondary care is highly relevant for protection of these patients against co-morbidities such as COVID-19 co-diagnosis: PCa patients are strongly predisposed to increased hospitalisation and mortality rates, for example, compared to patients with non-prostate genitourinary malignancies infected with COVID-19 [28].

## Making palliative care to the management of chronic disease

As demonstrated by current statistics of reactive medical services, biggest budgets are dedicated to the last year of the patients’ life in the PCa cohorts which is particularly unfortunate considering highly restricted life quality of patients in palliative care. In contrast, PPPM concepts intend to provide maximum efforts (intensified research, enrich diagnostic tools, increased financial input, etc.) to stabilise health condition of affected individuals at the initial care levels, thereby improving individual outcomes and ensuring significant socio-economic benefits to the society. In the transition phase to once fully implemented PPPM care, a big portion of affected individuals still will be provided with the reactive medical services and consequently will be predisposed to the progressing disease. Therefore, the motivation is to advance palliative care making it to the management of chronic disease. This task of tertiary care is highly ambitious and extraordinarily expensive, requiring an application of artificial intelligence (e.g. unsupervised machine learning) for the multi-parametric analysis adapting treatment algorithms to comprehensive individualised patient profiles, extending overall survival and improving individual outcomes [29]. One of the promising areas to learn from is the abscopal effects, when radiation therapy applied induces systemic antitumour effects outside of the irradiated field. Since the introduction of immune checkpoint inhibition, corresponding reports on abscopal effects appear increasingly in the literature and may allow for identifying clinically relevant patterns for diagnostic and treatment purposes [30].

Finally, application of natural compounds based on flavonoids and their nano-technologically created derivatives demonstrate great clinical potential, due to their immune-modulating and drug-sensitising effects applicable to primary, secondary and tertiary care. Their ability to increase sensitivity of cancer cells and to reverse cancer resistance against anti-cancer therapies is a promising approach complementary to other therapeutic modalities applied [31].

## Data Availability

Not applicable
